# Critical Care Admissions following Total Laryngectomy: Is It Time to Change Our Practice?

**DOI:** 10.1155/2016/8107892

**Published:** 2016-09-26

**Authors:** Hussein Walijee, Alexandria Morgan, Bethan Gibson, Sandeep Berry, Ali Jaffery

**Affiliations:** ^1^Department of Otorhinolaryngology, Royal Glamorgan Hospital, Llantrisant CF72 8XR, UK; ^2^Department of Anaesthesia and Critical Care, Royal Glamorgan Hospital, Llantrisant CF72 8XR, UK

## Abstract

Critical Care Unit (CCU) beds are a limited resource and in increasing demand. Studies have shown that complex head and neck patients can be safely managed on a ward setting given the appropriate staffing and support. This retrospective case series aims to quantify the CCU care received by patients following total laryngectomy (TL) at a District General Hospital (DGH) and compare patient outcomes in an attempt to inform current practice. Data relating to TL were collected over a 5-year period from 1st January 2010 to 31st December 2015. A total of 22 patients were included. All patients were admitted to CCU postoperatively for an average length of stay of 25.5 hours. 95% of these patients were admitted to CCU for the purpose of close monitoring only, not requiring any active treatment prior to discharge to the ward. 73% of total complications were encountered after the first 24 hours postoperatively at which point patients had been stepped down to ward care. Avoiding the use of CCU beds and instead providing the appropriate level of care on the ward would result in a potential cost saving of approximately £8,000 with no influence on patient morbidity and mortality.

## 1. Introduction

Limited hospital financial and personnel resources coupled with the need for established evidence-based clinical practice have forced surgeons and anaesthetists to consider appropriate triage for the immediate postoperative management of patients who have been subjected to major head and neck surgery. Poor nutritional status, diabetes mellitus, and chronic diseases of the respiratory and cardiovascular systems as a result of long standing alcohol and tobacco use are factors frequently encountered in patients with head and neck cancers [1]. Neoadjuvant radiotherapy, extensive surgery, and flap reconstruction further influence the postoperative recovery and risk of complications [2].

It is common practice in centres worldwide to admit patients to Critical Care Unit (CCU) for 24 hours following major head and neck surgery in order to allow for airway oedema to stabilise, facilitating safe extubation prior to stepping down to the ward. In the case of patients undergoing TL, a cuffed tracheostomy tube is placed into the newly formed stoma to protect the airway; however, admission to CCU remains routine practice in many hospitals including ours. The drawbacks of such practice may include cancellation of operations due to lack of CCU beds, the intrinsic expense of critical care, and lack of continuation of care for patients [3].

Multiple studies [3–6] have provided evidence on the safety of nursing head and neck cancer patients on a specialised ward with adequately trained nursing staff with no evidence of increase in the risk of morbidity or mortality.

Patients undergoing a total laryngectomy (TL) at the Royal Glamorgan Hospital (RGH), a District General Hospital (DGH) in South Wales, UK, are routinely admitted to level 2 (High Dependency Unit, HDU) or level 3 (Intensive Therapy Unit, ITU) postoperatively depending on patient comorbidity and bed availability. Following 24 hours of close monitoring, they are transferred to the ENT ward (level 0) [7].

This case series aims to quantify CCU care received by patients following TL at a DGH and compare morbidity and mortality rates, in an attempt to inform current practice.

 Classifications of levels of care (adapted from [7]) are as follows:

Level  0: patients that can be managed on a normal ward care in an acute hospital

Level  1: patients at risk of their condition deteriorating, who can be managed on an acute ward with additional advice and support from the CCU team

Level  2: patients requiring more detailed observation or intervention including support for a single failing organ system and those “stepping down” from higher levels of care

Level  3: patients requiring respiratory support and/or support of at least two organ systems. This level includes all complex patients requiring support for multiorgan failure

## 2. Materials and Methods

Anonymised data were collected retrospectively for those undergoing TL, coded E2910 at RGH between 1st March 2010 and 31st January 2015. As this was extracted from routinely collected data, formal ethical approval was not required. This study was conducted in compliance with trust audit and data protection protocols. Head and neck centres across Wales were invited to complete an online (see Appendix) and telephone survey to obtain data regarding postoperative level of care for TL patients.

The patients' operative risk as assessed prior to the procedure was characterized by the American Society of Anesthesiology (ASA) grade. Intraoperative data collected were the procedure type, operating time, estimated blood loss, and documented complications. The patient's vital signs and blood results were obtained from the computerised CCU records and patient notes to allow calculation of POSSUM and P-POSSUM. All patients were graded with an operative severity of “major” as per Griffiths et al. [8] (Table 1(a)).

Each patient was allocated a score depending on severity (1, 2, 4, or 8) for each of the 12 physiological parameters (PS) and 6 operative parameters (OS) as shown in Table 1(b), reproduced from Copelands work [9] on POSSUM (Physiological and Operative Severity Score for the enUmeration of Mortality and morbidity). From these values predicted morbidity and mortality were calculated using formulae below

POSSUM morbidity % risk (*R*) is

(1) $$\begin{array}{c} \ln\left[\;\left(\;\frac{R}{\left(1-R\right)}\;\right)\;\right]=-5.91+\left(\;0.16\times PS\;\right)+\left(\;0.19\times OS\;\right). \end{array}$$

POSSUM mortality % risk (*r*) is

(2) $$\begin{array}{c} \ln\left[\;\left(\;\frac{r}{\left(1-r\right)}\;\right)\;\right]=-7.04+\left(\;0.13\times PS\;\right)+\left(\;0.16\times OS\;\right). \end{array}$$

The P-POSSUM (Portsmouth POSSUM), a refinement of the original scoring system, utilises the same 18 physiological and operative parameters, but a different formula is applied to determine predicted mortality.

P-POSSUM mortality % risk (*P*) is

(3) ln⁡P1−P=−9.065+0.1692×PS+0.155×OS.

Details regarding complications encountered by patients were classified into surgical site complication, superficial infection, wound dehiscence, bleeding, myocardial infarction, pneumonia, fluid overload, renal failure, return to theatre, mechanical ventilation after surgery, ARDS, cardiac arrest, and death. Each complication, for the purpose of statistical analysis, was treated as a single episode (Table 3).

Statistical analyses were carried out using the Pearson correlation. A *p*-value of <0.05 was considered statistically significant. Data were analysed in MS Excel 2013 and IBM SPSS Statistics 20.

## 3. Results

From 2010 to 2015, 22 patients electively underwent TL with or without neck dissection and hemithyroidectomy. All patients had a primary closure immediately after resection. There were no emergency TL within this period. They ranged in age from 43 to 89 years (median 65 years), including 17 males and 5 females. No patients died during surgery and all patients were admitted to HDU postoperatively for close monitoring. The average length of the procedure was 7 hours and 56 minutes (SD 1 hour 21 mins).

Table 2 summarises the mean age, procedure length, ASA class, and POSSUM scores in relation to the operative procedure.

Patient 1 required invasive mechanical ventilation for a period of 6 hours on admission to HDU postoperatively following total laryngectomy + partial pharyngectomy + hemithyroidectomy + bilateral neck dissection (TLPPHT). The length of the surgery was 8 hrs and 4 mins and the patient was transferred back to the ward after a total HDU stay of 22 hours and 15 mins (ASA 3, P-POSSUM 1.7).

Patient 2 returned to theatre to arrest a haemorrhage encountered 6 hours after TLPPHT requiring invasive mechanical ventilation on return to ITU for 9 hours (length of CCU stay 46 hours, P-POSSUM 2.5, and ASA 3).

Bleeding occurred in two other patients between 7 and 30 days postoperatively. Patient 3 returned to theatre 10 days following TLPPHT due to intermittent chyle discharge from a wound dehiscence. The 72-year-old underwent a washout and repair of chyle leak and primary closure before being discharged to the ward. Two days later, the patient suffered from haematemesis, returning to theatre for a second time for repair of a defect in the internal jugular vein (IJV) and pharyngeal fistula. Three weeks postoperatively this patient underwent a debridement and pectoralis major flap reconstruction due to wound dehiscence (length of hospital stay 85 days, P-POSSUM 3.9, and ASA 3). Patient 4 suffered from a cardiac arrest thirteen days following TLPPHT after a myocardial infarction as a result of an estimated 3-litre haemorrhage. This patient was resuscitated with blood products and transferred to emergency theatres to control the haemorrhage originating in the right IJV and later moved to ITU for mechanical ventilation and inotropic support. The patient developed Acute Respiratory Distress Syndrome and died 3 days later (ASA 4, P-POSSUM 7.1).

Patient 5 was fluid overloaded in the immediate postoperative period and was treated effectively with intravenous diuretic and biochemical monitoring of renal function (length of HDU stay 20 hours 10 mins, P-POSSUM 2.8, and ASA 3).

Three patients developed pneumonia. Two within the first 24 hours: both patients 6 and 7 had undergone TLPPHT and were found to have increased oxygen and suction requirements as well as radiological evidence of consolidation (mean length of HDU stay 23 hours 45 mins, P-POSSUM 9.2, and ASA 3). The third, patient 8, developed pneumonia 6 days following TLPPHT and recovered following treatment with intravenous antibiotics (length of HDU stay 16 hours 15 mins, P-POSSUM 3.1, and ASA 3).

Patient 9 was admitted to ITU postoperatively for level 2 care (P-POSSUM 2, ASA 2, and length of stay in CCU 23 hrs and 50 mins) and patient 10 cancelled due to lack of HDU bed.

Patient 11, a 64-year-old with previous neck irradiation, had a pharyngeal leak 9 days following TL. He was successfully managed conservatively and began oral feed at postoperative day 14 (ASA 3, P-POSSUM 0.8, and length of HDU stay 22 hours). The decision to transfer this patient to the ward was made at 16 hours; however, due to a lack of bed-space on the ENT ward, the transfer was delayed. The mean delay in discharge amongst the 22 patients was 3 hrs and 10 mins. The average length of CCU stay for the 22 patients was 25 hours and 27 minutes.

Head and neck centres across Wales were invited to complete a telephone and online questionnaire survey (see Appendix) regarding postoperative practice for patients following TL.  Table 4 summarises these results.

Pearson correlation analysis between the number of complications per patient and individual P-POSSUM mortality and POSSUM morbidity risk scores did not show a significant relationship (*p* = 0.648 and *p* = 0.362, resp.).

## 4. Discussion

This case series investigates the morbidity and mortality amongst a cohort of patients undergoing TL with or without additional procedures. It focuses on the incidence and timescale in the development of postoperative complications whilst comparing the outcomes to P-POSSUM mortality risk. The linear average for P-POSSUM predicted mortality was 6.68% which on a cohort of 22 patients is 1.5 predicted deaths. The observed mortality of 1 echoes previous studies [8, 10] suggesting that P-POSSUM overpredicts mortality rates.

Copeland et al.'s POSSUM [9] and its refined P-POSSUM version by Prytherch et al. [11] were primarily validated on a general surgical population. Following its publication, large cohorts of patients have been used to validate variations of POSSUM for specific surgical groups including Cr-POSSUM for colorectal surgery [12] and V-POSSUM for vascular surgery [13], deemed more sensitive and specific for predicting patient outcomes following these operations.

There was no significant correlation between the number of complications with POSSUM (*p* = 0.362) and that with P-POSSUM (*p* = 0.648) within our patient cohort. Griffiths et al. [8] concluded, based on a sample of 301 head and neck patients, that POSSUM and P-POSSUM scoring did not appear to have relevance in predicting mortality. Variables such as “peritoneal soiling” and the “Glasgow Coma Scale” designed for a general surgical population would not be relevant for head and neck patients. In an attempt to make POSSUM applicable to the head and neck population, Griffiths et al. [8] suggested inclusion of radiotherapy and previous surgery as variables.

The Royal College of Surgeons in England Working Group [14] recommended that patients with a predicted P-POSSUM mortality of ≥ 10% should be admitted to a critical care location postoperatively. Amongst the 22 patients, 3 patients would have fallen within this category and therefore would have required admission to critical care based on these recommendations.

Cardiopulmonary Exercise Testing (CPET) has become increasingly popular for preoperative assessment as it represents a noninvasive simulation of the requirements of major surgery. CPET has been deemed a powerful diagnostic and prognostic tool for a variety of medical disorders, including coronary artery disease, cardiac failure, and restrictive and obstructive pulmonary disease. It can be used as a valuable resource for providing detailed evaluation of patients' functional status before major surgery [15, 16].

Cost control and efficient use of resources are becoming increasingly vital in modern medicine, but it remains essential that cost saving measures should not adversely affect the quality of patient care. Some of the factors influencing this challenge are the changing patient demographics and the high cost of medical equipment and technology in addition to rising patient and public expectation of higher standards of surgical care.

Inappropriate utilisation of critical care facilities has been discussed most notably by Knaus et al. [17]. The authors found that, during 49% of admissions and 65% of the nursing shifts, the emphasis was on close nursing care and observations and not intensive treatment. They also reported that 86% of patients admitted for close monitoring did not require active treatment prior to discharge.

In our case series, one patient required invasive mechanical ventilation immediately postoperatively and was therefore appropriately placed to be managed in CCU. 21 patients (95%) admitted to HDU for close monitoring did not require any intervention that could not be delivered on ENT ward.

In 1999, Godden et al. [3] demonstrated that it was safe to care for head and neck patients outside the ITU, provided it was done in an appropriate environment with adequately trained nursing staff, close consultant support, and sufficient throughput to provide experiential training. They found little difference in the general complications between the group of patients nursed on the ward with “special care for 48 hours” and those admitted directly to ITU for 24 hours before being transferred to the ward for a further “24 hours of special nursing.”

Hanna et al. [6] developed a clinical pathway for patients undergoing TL and assessed its impact on their outcomes. Patients were transferred to ITU only for specific indications requiring an increased level of care such as respiratory or haemodynamic instability. This protocol did not result in increased morbidity or mortality amongst “pathway patients” compared to “prepathway patients.” They reported a cost saving of 22.5% in the pathway group. This supported the findings from Husbands et al. [18] who introduced care pathways to the management of head and neck surgical patients and found significant reductions in hospital costs as well as shorter lengths of hospital stay.

Chang et al. [19] prospective study involving 304 patients following radical cystectomy assessed the difference in outcomes between patients who were managed on the ward and those requiring intensive care monitoring. 93.7% of their patients were managed safely on a surgical ward and did not require CCU. They stress the importance of preoperative optimisation, early consultation with specialists, and working closely with intensivists during the perioperative period.

Close monitoring on an ENT specialist ward within the immediate postoperative period would involve 1 : 1 care provided by a trained ENT nurse for 24 hours. The aim of specialist nursing care would include hourly monitoring of urine output, respiratory rate, blood pressure, and pulse; care of the laryngectomy stoma with humidified air, oxygen, and suction with continuous monitoring of oxygen saturations; and care of the wound with 2 hourly checks for bleeding or haematoma, administering intravenous fluids, and standard care of pressure areas which would involve turning the patient and palpating the skin every 8 to 24 hours [20]. Pain management is achieved with a patient controlled analgesia supplemented with paracetamol. This would typically provide adequate pain relief whilst reducing the risk of respiratory depression. It is impossible to avoid all postoperative complications; however, in order for postoperative sequelae to be minimised, close monitoring is essential, enabling rapid recognition and treatment of ensuing complications.

By avoiding the use of CCU beds and instead ensuring that the nurses in the head and neck ward are adequately trained to manage these patients, it is likely that their recovery and discharge are expedited [3]. Other than the four patients—three with a P-POSSUM > 10% and one requiring active intervention in CCU—18 patients from the case series may have been nursed on ENT specialist ward with 1 : 1 nursing for the first 24 postoperative hours. The cost per night of a bed in the ENT ward is £290, and the additional cost of a specialist ENT nurse for the 24-hour period is an estimated £290 within the trust. Based on the throughput for the period of the study this would cost a total of £10,440 which represents a minimum saving of £7,848. This is because CCU is an expensive commodity costing £1016 in HDU and £2,661 in ITU per day.

In Wales there was an average of 3.2 critical care beds per 100,000 people in 2014, lower than the number available for the population in the rest of the UK. The Welsh government has recommended that units run at an average occupancy of 65–70%; however, all units in Wales report occupancy rates greater than 80%, with many often operating at over 100% occupancy at times [21]. It is suggested that occupancy of ≥80% is “likely to result in nonclinical transfers and failure of admittance in a timely manner with associated morbidity and mortality” [22].

In our case series, one patient was admitted to ITU postoperatively for level 2 care and another was cancelled due to lack of a HDU bed. Not using CCU for these patients would mean that operations are not dependent on the availability of a bed and therefore not cancelled on the day of operation, ultimately benefiting the patients and their families who will be spared the anxiety that this causes. Benefits to the hospital are harder to estimate but would include loss of revenue if operating lists were cancelled. The Institute for Innovation and Improvement [23] estimates running costs for an average operating theatre at £1,200 per hour; therefore, greater efficiency and careful use of resources are paramount. Critical care beds may become “blocked” when patients are unable to be discharged due to a lack of ward beds. If a shortage of critical care beds were to cause an increase in cancellation rates of major elective surgery, hospitals may fail to meet targets [24].

The limitations of our study include its retrospective methodology and possible selection bias inevitable in an operative series. It is difficult to predict whether the outcome of the patients within our case series would have differed had they not been admitted to HDU for the initial 24 hours due to the lack of a control group. The potential cost saving of £7,848 is therefore only theoretical. Further studies should aim to compare the outcomes of patients nursed on different levels of care within Wales and their cost implications.

The inconsistency of the levels of care for post-TL patients across Wales may be due to several factors: funding issues, critical care capacity and workload, expertise of nursing staff available, adequate throughput, and dedicated medical teams to care for the patient once on the ward. Several studies [3, 24] have shown that head and neck cancer surgery does not necessitate admission to critical care and therefore patients can be safely managed in a ward environment. There is no substitute for sound clinical judgement and decisions must be tailored to each individual. Undoubtedly certain patients require active intervention available only in critical care. However, the evidence suggests that admission to CCU can safely be the exception and not the norm.

## 5. Conclusion

Based on a small patient sample, the observed 30-day mortality was encountered on the fifteenth postoperative day, well beyond the first 24 hours following discharge from CCU to the ward. Close monitoring and nursing care necessary within this period could be provided on a specialist ENT ward with appropriately trained one-to-one nurses. The authors would advocate a change towards ward-based care for postlaryngectomy patients and meticulous preoperative assessment to identify patients requiring CCU admission in order to reduce unplanned admissions.

Keeping appropriately selected patients out of CCU allows nursing staff more familiar with head and neck cancer patients to care for them and helps maintain an “esprit de corps” amongst ENT nurses and surgeons, whilst freeing up critical care beds for those in most clinical need [5].

## Figures and Tables

**(a):** 

Operative severity	Score	Operation type
Minor	1	Endoscopy, tracheostomy, excision submandibular gland/lymph node
Moderate	2	Parotidectomy, thyroidectomy, neck dissection
Major	4	Laryngectomy, pharyngectomy
Complex major	8	Free flaps, pedicled flaps

**(b):** 

Physiological parameters	Operative parameters
Age (years)	Operative severity
Cardiac signs and chest radiograph	Multiple procedures
Respiratory history and chest radiograph	Total blood loss
Systolic blood pressure (mmHg)	Peritoneal soiling
Pulse (beats/min)	Presence of malignancy
Glasgow Coma Scale (GCS)	Mode of surgery
Haemoglobin (g/100 mL)	
White cell count (×10^12^/L)	
Urea (mmol/L)	
Sodium (mmol/L)	
Potassium (mmol/L)	
Electrocardiogram	

**Table 2:** Mean age, length of procedure, ASA class, and POSSUM scores in relation to operative procedure (standard deviation).

Surgery	*n*	Age	Length of procedure	ASA class				POSSUM		P-POSSUM mortality (%)
				I	II	III	IV	Morbidity (%)	Mortality (%)	
Total laryngectomy	1	64	4 hrs	0	0	1	0	95.1	3.1	0.8
Total laryngectomy unilateral neck dissection	1	73.55	8 hrs, 12 mins	0	0	1	0	17.7	57.7	31.5
Total laryngectomy + bilateral neck dissection	2	62.04 (9.36)	7 hrs, 25 mins (20 mins)	0	0	2	0	59.4 (3.7)	13.7 (1.6)	4.3 (0.4)
Total laryngectomy + partial pharyngectomy + hemithyroidectomy + bilateral neck dissection (TLPPHT)	18	65.07 (10.45)	8 hrs, 12 mins (15 mins)	0	4	11	3	57.8 (16.8)	16.4 (13.0)	5.9 (7.6)

Total	22	65.13 (9.8)	7 hrs, 56 mins (71 mins)	0	4	15	3	57.7 (19.2)	17.4 (15.1)	6.7 (8.9)

**Table 3:** Complications postoperatively divided into the first 24 hours, 1–7 days, and 8–30 days.

Complication	Time after surgery														
		<24 hours					1–7 days					8–30 days			
		*n*	%	P-POSSUM average	Average time spent in HDU		*n*	%	P-POSSUM average	Average time spent in HDU		*n*	%	P-POSSUM average	Average time spent in HDU
Overall complications		6	27	4.7	21:40		3	14	2.9	14:00		14	59	5.8	17:15
Surgical site complication		—	—	—	—		1	4	2.6	14:20		1	4	0.8	22
Superficial infection		—	—	—	—		—	—	—	—		2	8	2	15:20
Wound dehiscence		—	—	—	—		—	—	—	—		3	12	13.1	19:07
Bleeding		1	4	2.5	22:00		—	—	—	—		2	8	5	19:05
Myocardial infarction		—	—	—	—		—	—	—	—		1	4	7.1	12:00
Pneumonia		2	8	9.2	23:45		1	4	3.1	16:15		—	—	—	—
Fluid overload		1	4	2.8	20:10		—	—	—	—		—	—	—	—
Renal failure		—	—	—	—		1	4	6	11:30		—	—	—	—
Return to theatre		1	4	2.5	22:00		—	—	—	—		2	8	5.5	23:20
Mechanical ventilation after surgery		1	4	1.7	18:00		—	—	—	—		—	—	—	—
ARDS		—	—	—	—		—	—	—	—		1	4	7.1	12:00
Cardiac arrest		—	—	—	—		—	—	—	—		1	4	7.1	12.00
Death		—	—	—	—		—	—	—	—		1	4	7.1	12:00

**Table 4:** Variation of postoperative discharge destination across Head and Neck centres in Wales.

Head and Neck centre in Wales		Postoperative level of care
Location	Hospital	
Cardiff	University Hospital of Wales	Level 0 Level 2 (flap reconstruction/ASA 4)

Newport	Royal Gwent Hospital	Level 0 Level 2 (flap reconstruction)

Bridgend	Princess of Wales Hospital	Level 0 + one-to-one nursing care 24 hours Level 2 (anaesthetic concerns)

Swansea	Singleton Hospital	Level 2 (24 hours)

Carmarthen	Glangwili General Hospital	Level 0 + one-to-one nursing care 24 hours

Rhyl	Glan Clwyd Hospital	Level 2 (24 hours)

Llantrisant	Royal Glamorgan Hospital	Level 2 (24 hours)

## Appendix

### Online Survey

1. Which NHS trust are you currently employed in?
2. Which department do you currently work in?
3. Current grade
  □ Foundation Year 1-2 Trainee
  □ Core Surgical Trainee year 1-2
  □ Registrar
  □ Staff grade doctor
  □ Associate Specialist
  □ Consultant
4. Are you aware of the departmental practice regarding the location laryngectomy patients are transferred to immediately post-op for the first 24 hours?
5. Where are patients routinely cared for during the first 24 hours following a laryngectomy within your NHS trust?
6. Are you aware of any guidelines used in the department to determine where patients should be cared for during the first 24 hours post-laryngectomy?
7. If your answer to Qs (6) above is yes, are these guidelines based on:
  □ Consultant preference
  □ Number of patient co-morbidities
  □ Pre-operative P-POSSUM mortality risk
  □ Other (please specify)
8. Any other comments

## Additional Points

*Summary. *(i) Historically major operations have not required an automatic admission to CCU. The majority of postoperative patients admitted for close monitoring at the study centre did not require active treatment prior to discharge to the ward. (ii) Multiple studies have shown that it is safe to manage complex head and neck patients on a ward setting given the appropriate staffing and support. This in turn would reduce demand on CCU beds and cancellation of elective surgery due to lack of CCU beds. (iii) The authors would advocate a change towards ward-based care for postlaryngectomy patients and meticulous preoperative assessment to identify patients requiring CCU admission in order to reduce unplanned admissions.
